# Digital wellness or digital dependency? a critical examination of mental health apps and their implications

**DOI:** 10.3389/fpsyt.2025.1581779

**Published:** 2025-04-03

**Authors:** Anithamol Babu, Akhil P. Joseph

**Affiliations:** ^1^ School of Social Work, Marian College Kuttikkanam Autonomous, Kuttikkanam, Kerala, India; ^2^ School of Social Work, Tata Institute of Social Sciences Guwahati Off-Campus, Jalukbari, India; ^3^ Department of Sociology & Social Work, Christ (Deemed to be University), Bengaluru, Karnataka, India

**Keywords:** digital mental health, algorithm-driven therapy, mental health apps, psychiatric care, regulatory oversight

The widespread integration of mental health applications marks a significant transformation in mental healthcare, but critical concerns remain about their clinical validity, ethical implications, and the risk of fostering digital dependence that may delay or replace necessary professional intervention (1). As the global prevalence of anxiety, depression, and stress-related disorders continues to rise, commercial platforms such as Calm, Headspace, and Wysa position themselves as accessible, cost-effective alternatives to conventional psychotherapy (2). While these interventions may offer transient symptomatic relief, their widespread assimilation into mainstream mental healthcare poses significant risks, as the lack of empirical validation on their long-term efficacy and clinical applicability may lead to ineffective or even harmful treatment outcomes, necessitating stronger clinical oversight and regulatory scrutiny (3). The predominance of algorithm-driven engagement over therapist-mediated interventions raises critical concerns about their role in contemporary mental health practice, particularly given evidence of algorithmic bias and data harms that reinforce pre-existing disparities by privileging certain user behaviors while marginalizing others (4). The opacity of proprietary algorithms further complicates efforts to assess whether these tools equitably serve diverse populations or inadvertently exacerbate mental health inequalities, ultimately undermining accessibility and clinical efficacy (5, 6). Consequently, a pivotal question emerges: Do these digital interventions meaningfully enhance psychological well-being, as some studies suggest, or do they foster an illusory sense of autonomy that may ultimately exacerbate underlying psychopathology, as indicated by research highlighting their limitations in addressing complex mental health needs (5, 7)?

Despite their accessibility, mental health applications, particularly AI-driven chatbots and self-guided therapy platforms, often adopt a reductionist approach to psychological distress, addressing surface-level symptoms while neglecting the intricate etiology of mental disorders (8). While these platforms extend access to self-guided meditation, cognitive behavioral therapy (CBT) modules, and AI-driven emotional support chatbots, their indiscriminate adoption risks fostering overreliance, dissuading individuals from pursuing professional mental health intervention, and delaying the initiation of evidence-based treatment (9). Such postponement may inadvertently precipitate poorer clinical outcomes, increasing susceptibility to chronic and treatment-resistant psychopathologies. Moreover, digital mental health interventions can foster a false sense of security, misleading individuals into believing they are receiving adequate care while their underlying conditions worsen due to the absence of timely in-person intervention, potentially leading to severe clinical deterioration and prolonged distress. This misplaced confidence in self-guided digital tools can delay necessary clinical treatment, exacerbating underlying conditions and reducing the likelihood of successful therapeutic outcomes (7, 10, 11).

While public-sector innovations in digital mental healthcare have significantly improved access to psychological support, the overreliance on behavioral reinforcement mechanisms -streaks, push notifications, and gamification elements- remains a pressing concern (12). These engagement-driven strategies, mirroring those used in social media addiction models, risk prioritizing user retention over genuine therapeutic benefit, fostering habitual app usage rather than meaningful psychological progress (13). These features, rooted in persuasive technology, prioritize engagement metrics over therapeutic efficacy, fostering compulsive digital behaviors that undermine self-regulation and blur the line between mental health support and digital dependency. Streak-based incentives in apps like Headspace and Calm promote habitual use over genuine improvement, while AI-driven chatbots such as Woebot simulate therapeutic conversations without the adaptability or depth of professional intervention, misleading users into perceiving them as viable substitutes for clinical care (14, 15). These strategies reinforce compulsive digital behaviors under the pretence of mental wellness by leveraging persuasive technology techniques that increase habitual app usage while reducing real-world social interactions, raising ethical concerns about their long-term psychological impact (16, 17). The persuasive technology embedded in these applications often mirrors mechanisms found in addictive digital platforms, such as variable rewards, push notifications, and streak-based incentives, which condition users to return habitually rather than engage meaningfully with their mental health. This manipulation of user behavior raises significant ethical concerns, as it blurs the line between promoting well-being and fostering digital dependency. Furthermore, the use of AI-driven nudges tailored to maximize engagement rather than therapeutic outcomes risks exacerbating psychological distress, particularly among vulnerable populations predisposed to compulsive digital behaviors. Rather than cultivating genuine self-regulation, such interventions risk engendering digital dependency, wherein users conflate app engagement with substantive psychological progress, reinforcing cyclical usage with limited therapeutic benefit (16, 17).

Moreover, one of the most persistent challenges facing digital mental health interventions is the issue of user retention, compounded by the fact that online user app ratings and the number of app downloads are inadequate predictors of an app’s quality in terms of user experience, professional or clinical assurance, and data privacy (18). Koh et al. (5) found that completion rates for digital mental health interventions are alarmingly low, with as few as 29.4% of young people completing the programs they begin. Most users abandon mental health applications soon after downloading due to app fatigue, unmet expectations, and usability concerns, reflecting design flaws that hinder sustained engagement (19, 20). The challenge of sustained engagement extends beyond individual user preferences to structural deficiencies in how digital interventions are designed and implemented. While improved user interface design may enhance engagement, it is insufficient to ensure sustained effectiveness; only systemic integration of digital mental health tools within established healthcare infrastructures can provide meaningful, long-term therapeutic outcomes (13, 21, 22).

Despite their technological sophistication, mental health applications fundamentally lack the cornerstone of mental health treatment: empathic, individualized human interaction (23). Psychological resilience and sustained recovery necessitate dynamic, therapist-directed interventions that adapt to a patient’s evolving psychopathology-an essential component absent in automated, algorithm-driven digital platforms (24). The autonomy offered by these applications fosters a misguided perception that mental health conditions can be self-managed, ultimately diminishing the perceived necessity of professional oversight, as evidenced by cases where individuals relying solely on digital interventions experienced worsening symptoms, delayed clinical treatment, or an increased risk of relapse (23, 24). The commercialization of digital mental health often prioritizes revenue generation and user engagement metrics over clinical effectiveness, as evidenced by the growing reliance on subscription-based models, data monetization through targeted advertising, and premium-tier access to essential mental health features, raising ethical concerns about accessibility and equity. Without stronger public health-driven solutions, including regulatory oversight and transparent, ethical guidelines, digital mental health platforms risk deepening existing disparities by limiting access to high-quality psychological support for economically disadvantaged populations (25). Despite regulatory efforts such as the European Union’s General Data Protection Regulation (GDPR), enforcement remains inconsistent, as evidenced by cases like the 2021 BetterHelp controversy, where user data was shared with advertisers without proper consent, and multiple mental health apps failed to meet basic data security and clinical validation standards. These failures have led to consumer distrust, potential privacy violations, and continued reliance on unverified applications, highlighting the urgent need for more vigorous regulatory enforcement, third-party audits, and standardized clinical assessments to ensure digital mental health tools meet ethical and professional healthcare standards. Governments should introduce funding incentives for developers prioritizing patient safety and evidence-based methodologies to foster a regulatory ecosystem that upholds ethical and clinically sound digital mental health solutions (26). The commodification of mental healthcare deepens systemic disparities, limiting access to quality psychological support for those unable to afford premium services. However, government-subsidized teletherapy services and nonprofit initiatives, such as Australia’s Head to Health and Mind in the UK, offer free, evidence-based digital resources integrated with professional care, ensuring equitable mental health access without reliance on monetized engagement strategies (27, 28). Platforms like BetterHelp and Talkspace employ tiered pricing models that restrict vital therapeutic features to premium subscribers, prioritizing profitability over accessibility and exacerbating socioeconomic disparities in mental health care (29, 30).

Without structured integration and therapist oversight, digital mental health applications risk becoming temporary solutions rather than sustainable therapeutic tools, requiring collaboration between regulatory bodies, clinical institutions, and developers to standardize evidence-based protocols. Hybrid models, such as stepped-care approaches and telepsychiatry programs, illustrate how integrating digital interventions with human-centered mental health care can improve accessibility and efficacy while mitigating the risks of overreliance on technology. However, the mere adoption of digital mental health tools is insufficient; national healthcare systems must implement stringent regulatory and clinical validation frameworks to ensure their effectiveness and safety. The NHS (United Kingdom) has developed the Digital Health Assessment Framework (DHAF) to accredit digital mental health applications based on safety, usability, and clinical effectiveness, while Ayushman Bharat’s (India) Health and Wellness Centers (HWCs) have introduced telepsychiatry services to expand access to mental health care in rural areas (31, 32). Without coordinated efforts between policymakers, mental health professionals, and technology developers, digital interventions will remain fragmented, exacerbating systemic inequities in mental healthcare and limiting their long-term impact. Governments must enforce clinician-led implementation, foster cross-sector collaboration, and establish sustainable funding models to ensure digital mental health tools serve as evidence-based, equitable, and effective complements to traditional care rather than commercialized, unregulated substitutes that undermine professional oversight. Sustainable funding through insurance reimbursement and structured regulatory oversight is essential to integrating digital mental health tools into mainstream care. Telepsychiatry’s collaborative care models, which combine AI-driven assessments with therapist-led interventions, and stepped-care approaches like the IAPT (Improving Access to Psychological Therapies) program in the UK, which uses digital CBT modules as a preliminary step before escalating cases to in-person therapy, exemplify how technology can enhance accessibility while maintaining clinical efficacy, particularly for individuals with limited access to mental health professionals (33). These models exemplify how technology can enhance accessibility while maintaining clinical efficacy, thereby reducing the likelihood of overreliance on digital interventions alone.

However, despite the exponential rise of mental health apps, only a negligible fraction has been subjected to rigorous evaluation and accreditation by authoritative bodies such as the Organisation for the Review of Care and Health Apps (ORCHA) or the M-Health Index and Navigation Database (MIND). ORCHA (34), a UK-based regulatory entity, evaluates mental health apps across essential parameters such as user experience, data privacy, and clinical assurance; however, with over 350,000 digital healthcare products available, of which 85% fall below established quality thresholds and only 20% meet safety standards, the challenge extends beyond availability to the urgent need for healthcare professionals to have reliable tools for identifying clinically effective digital solutions. This overwhelming lack of regulation raises a pressing question: how can professionals and users address an unchecked and chaotic landscape dominated by unverified and potentially harmful applications to ensure safe and effective mental health support?

Strengthening regulatory oversight requires mandatory certification for mental health apps, ensuring rigorous clinical validation before market release through established frameworks such as Food and Drug Administration (FDA) approvals for digital therapeutics in the United States, Conformité Européenne (CE) marking in Europe, or accreditation by the Organisation for the Review of Care and Health Apps (ORCHA) in the United Kingdom. Independent audits must be enforced to ensure transparency in data usage, algorithmic fairness, and ethical compliance, particularly in response to growing concerns over passive data collection, AI-driven discrimination, and privacy breaches in unregulated digital health solutions. Industry-led initiatives, such as standardized rating systems and incentives for collaboration between developers and mental health professionals, can further align digital solutions with evidence-based therapeutic principles, fostering a safer and more reliable mental healthcare framework. Similarly, Camacho et al. (35) conducted an extensive review of 578 mental health apps indexed in MIND across 105 dimensions, exposing a systemic deficiency in clinically innovative features and widespread privacy vulnerabilities that place users at significant risk. These findings reveal an alarming gap in regulatory oversight, reinforcing the urgency for stringent accreditation processes to ensure that digital mental health solutions adhere to established clinical and ethical standards. Without stringent regulatory oversight and standardized clinical validation, mental health applications pose significant ethical risks, including excessive surveillance, biometric monitoring, and AI-driven discrimination, concerns that are further underscored by failures in enforcement, such as the 2021 BetterHelp controversy and widespread data security lapses in mental health apps. These regulatory shortcomings highlight the need for comprehensive frameworks that mandate user consent, independent audits, and stringent accreditation processes to ensure ethical and clinically sound digital interventions. Current regulations remain inadequate, as most jurisdictions lack clear policies on passive data collection, algorithmic transparency, and the ethical use of biometric data in mental health interventions. To address these gaps, policymakers must implement mandatory user consent protocols, enforce independent audits for algorithmic fairness, and establish strict limitations on biometric data tracking to safeguard privacy and prevent discriminatory outcomes. Without these essential safeguards, digital mental health interventions will continue prioritizing commercial expansion over patient safety and scientific integrity, necessitating urgent systemic intervention to align technological advancements with ethical and professional healthcare standards.

Rigorous investigations should inform the establishment of robust, evidence-driven strategies to seamlessly integrate these tools into comprehensive, clinically validated mental health frameworks. Implementation strategies must mandate third-party audits to ensure algorithmic transparency, require therapist-led oversight for AI-driven interventions, and introduce interdisciplinary training programs to equip mental health professionals with the technical competencies needed to responsibly incorporate digital tools within established therapeutic models. Without these essential measures, digital mental health solutions will remain a fragmented, underregulated sector incapable of delivering meaningful, long-term psychological support. Furthermore, empirical research must prioritize large-scale longitudinal studies assessing the impact of digital interventions on treatment outcomes, relapse rates, and therapeutic adherence. By fostering a research-driven, ethically regulated approach to digital mental health, the field can ensure that mental health applications serve as beneficial adjuncts rather than substitutes for professional mental healthcare. Concrete disclaimers and pop-up messages should be mandated within these applications to explicitly inform users that they are not a replacement for professional mental health support. Moreover, these platforms should mandate the integration of a built-in feature that ensures immediate, seamless access to free and paid (public and private) mental health services, ensuring users can seamlessly connect with nearby professionals when necessary. Beyond accessibility, further empirical research is imperative to evaluate the real-world effectiveness of such integrations, examining user engagement patterns, intervention outcomes, and the long-term impact of digital mental health tools on psychological well-being. While mental health apps themselves are not inherently problematic, the challenge arises when users perceive them as standalone solutions rather than supplementary tools within a broader therapeutic framework. Future studies must consolidate research efforts by rigorously evaluating digital interventions through randomized controlled trials (RCTs), real-world effectiveness studies, and qualitative assessments to ensure they function as effective complements rather than inadequate substitutes for professional mental healthcare. These approaches would help determine whether digital interventions serve as effective complements to traditional mental healthcare or risk becoming inadequate substitutes.
